# Supplementary material for: Monitoring the ‘diabetes epidemic’: A framing analysis of United Kingdom print news 1993-2013
Source: PLoS One. 2020 Jan 17;15(1):e0225794. doi: 10.1371/journal.pone.0225794 (PMC6968867; doi:10.1371/journal.pone.0225794)
Supplement: Attachment — Submitted filename: PLOSONE_Diabetes media framing Review Sept 2019.docx [file pone.0225794.s001.docx]

This paper reports on a longitudinal analysis of how the ‘diabetes epidemic’ dialogue has played out in UK newspapers over a 20-year period. While this is an interesting and novel study, which has been well situated within the literature, there are some concerns around the methods and the results that I believe need to be addressed prior to publication.

**Introduction**

Main text, pages 5-6: You have touched on the issue that diabetes is a complex disease for which we do not know the exact causes, but, given the focus of your analysis on how the causes/risk factors for diabetes are presented, I think it would strengthen your manuscript to include a more detailed discussion of how the evidence base and our understanding of the factors associated with diabetes have shifted over the 20 years that you selected for your sample – this would allow for a more nuanced understanding of the shifts in discourse – for example, to what extent do some of the findings represent new understandings about diabetes (I noted for example that a number of your quotes included experts commenting on diabetes)?

Page 3, line 63: “…constructed in public imagination” – I suggest changing to “constructed in the media” or similar given that the focus of your analysis is only on the content of news media and not how this is interpreted by publics (construction of issues within the ‘public imagination’ will be the result of an interplay between the content of the message and how it is received)

Page 4, line 86: What is meant by the term ‘Super Value’ in reference to health? This needs explanation for those not familiar with the term

Page 5, line 105: “Some authors point out that some evidence… was overstated” – can you be more specific here about what evidence is thought to have been overstated?

Page 7, line 162: Here and in other places throughout the manuscript you have used the term ‘fatness’ instead of obesity, and these terms seem to be used interchangeably throughout. Is there a particular reason for this word choice? If not I would suggest simply using the more widely used (and less ‘loaded’) term ‘obesity’ throughout

**Materials and methods**

Page 8, line 185: “databases – name them – from the early 1990’s” – I assume that the names of the databases should have been inserted here?

Page 8, line 188: Stated that The Sun newspaper was included, but Table 1 lists the Daily Mail – please clarify which of these was included in the sample. Also stated that ‘not having a tabloid’ was a limitation – The Sun is considered to be a tabloid in the UK, as is the Daily Mail (as you state in Table 1 where you refer to it as mid-market tabloid)

Page 8, Line 191: Can you clarify which papers in your sample draw from Associated Press to see how much overlap there is likely to be within your included sample, as well as the overlap with the papers that weren’t included

Page 9: There is insufficient discussion of how the combinations of keywords were selected. In particular, I note from the Figure that at no point in the search strategy was the term ‘diabetes’ entered on it’s own, without being linked with obesity and/or epidemic. This is a potential problem when it comes to discussing the number of papers discussing diabetes as an epidemic or in relation to obesity in your sample, as you don’t have the sample of papers that talk about diabetes in the absence of these terms, which would be your denominator

Figure 1 Search Strategy: Missing number of papers identified in 2013, and needs to state which search fields were used – title, full text, excerpt?

Page 9: At what point in 2013 was the search performed? Do you have a complete 12 months of data for this year? This is important in considering how the number of articles changed across the years.

Page 9: Important information on the data handling process appears to be missing:

- How did you get from the 4280 articles originally identified to the 51, 119 and 324 papers across your 3 sample years?
- How many articles were identified in each database? How did you deal with duplicates across Factiva and Lexis Nexis (and why did you search in both databases if they both contain the same newspapers?)?
- Was the selection of articles for each of the years done automatically (e.g. by selecting an option in the database just to download articles from those years), or manually by going through and selecting all from those years?
- Was there a screening process to include/exclude papers according to a set of criteria? If so what were these criteria, did you review the full article text or just an excerpt, who did the screening and what reliability processes were in place (e.g. second person checking a subsample)?
- Were all records imported into NVivo and handled exclusively in NVivo or were there other data handling procedures?

Page 10, line 225: Stated that studies in this area have used thematic, CD and framing analysis – please provide references to key studies here for referecen

Page 10, line 227: Stated that “Using a method different to those used so far was thought to extent and complement…” but then you go on to say you used thematic and framing analysis – both of which you previously stated are common approaches in this area. Please clarify your meaning here.

Page 11, lines 241-243: Is the statement about piloting of computerised coding frame relevant to your methods? It seems unnecessary and I would suggest cutting for brevity

Page 12 Lines 255-261: More detail needed around the process of thematic analysis. Was this performed just by one author (and which one)? Was there any discussion process with other researchers to refine/explore interpretations? Was there an iterative process to coding?

Page 12, lines 263 – 267: More detail needed around the framing analysis:

- How did the researchers come up with/decide on the three frames? Was previous research consulted here? To what extent did these emerge from the inductive process?
- Need to define the various frames (the four from Entman and the three identified here) and provide examples to make it clear how these were applied (this could be in supplementary materials if needed).
- Who performed the framing analysis? Just one author? Did a second person check for consistency?

Page 13, lines 278-280: Stated that the researcher considered and cross checked the initial findings – was there any refinement here as a result of checking? Any discussions with the wider research team?

**Results**

Page 13, first paragraph of results: Would have been useful to provide a high level overview here, including the number of papers across each of the three sample years, and perhaps the key trends that you saw that you will unpack further. This would be more informative than simply explaining how the results are structured

One thing that for me was missing from the findings was a consideration of the frequency with which diabetes was actually discussed in terms of an ‘epidemic’ across the years of the sample – how often was this term actually used?

Page 13, lines 285-286: Need more detail to explain the content of the tables – state that shading refers to salience – but not clear if darker means ‘more salient’ or how salience was judged – this needs to be explained somewhere. Would perhaps be helpful to expand these tables to include an example in each cell.

Page 13, lines 300-301: Stated that small sample in 1993 suggests that diabetes was not as newsworthy – but related to my point above, that without a search term of ‘diabetes’ on its own (not in conjunction with obesity or epidemic) you can’t really make this statement – there may well have been articles in 1993 that talked about diabetes but not in the ‘linked’ way that your search terms targeted and that therefore may have been missed by your search.

Page 16, lines 353-356: This paragraph on medical framing may need some nuancing to make it clear why this falls into the ‘modifiable’ rather that ‘non-modifiable’ risk factors

In a number of the quotes that you use to support your descriptions of ‘lifestyle/behavioural framings’ of diabetes in the 2001 and 2013 samples, the data doesn’t actually explicitly mention lifestyle or behaviour and this seems to be implied by the author. For example, lines 363-368 the description focuses on role of lifestyle but the quote only mentions obesity as a cause of T2DM, without reference to lifestyle or behaviour. Similarly, the quote on page 20, lines 472-474 doesn’t mention lifestyle but is used in apparent support of the statement that there was a perception that diabetes and obesity can be addressed through lifestyle

Page 16, lines 370-374: This seems to pretty much just repeat what the description and quote in the previous lines was saying

Throughout the results it is not always clear which framings the data you are talking about fall into. It would perhaps be useful to have a few sentences at the start of the section for each year just summarising the key patterns in the data that are represented in the figure, before delving down into more detail and examples.

I would also perhaps have liked to see a reflection on whether there were differences across the three kinds of newspapers in how they portrayed diabetes?

Page 19, line 433-434: Stated that “diabetes increasingly presented as preventable through individual action and choice *alone*” – but the quotes that you present suggest a more nuanced understanding – i.e. that these things *reduce* risk, not that there are the *only* thing to do.

**Discussion**

Findings are situated well within the broader context and literature although, I would expect to see some consideration of the limitations of the study here.
